# miR-24 in diabetes

**DOI:** 10.18632/oncotarget.4795

**Published:** 2015-07-09

**Authors:** Yaozu Xiang

**Affiliations:** Yale University, School of Medicine, New Haven, CT, USA

Diabetes are common diseases threatening health worldwide. Over the past several years it has become clear that alterations in the expression of microRNA (miRNA) genes contribute to the pathogenesis of diabetes [1, 2]. miRNAs are small (~22 nt) regulatory RNA molecules that functionally modulate the activity of specific mRNA targets involving in a wide range of physiologic and pathologic processes [3]. miRNA expression profiling of diabetes has identified signatures associated with diagnosis, progression, prognosis and response to treatment [1]. Here, we focus on one specific miRNA: miR-24, which is involved in both diabetes and diabetic complications.

miR-24 is highly conserved in various species and is clustered with miR-23 and miR-27 on human chromosome 9 (miR-24-1) and 19 (miR-24-2). Both miR-24-1 and miR-24-2 generate the same mature product: miR-24. The levels of miR-24 in plasma of diabetes were significantly lower than that in healthy controls [4]. Notably, plasma levels of miR-24 inversely correlated with HbA1c [4]. miR-24 was highly expressed in the lungs, heart, pancreases, and kidneys but poorly expressed in the liver and muscle. Consistent with human observations, miR-24 levels were decreased 2-3 fold in STZ-induced mice and db/db diabetic mice lungs, and 5-fold in db/db mice plasma [4]. *In vitro* cultured cell line analyses indicate miR-24 is top highly expressed in endothelial cells and beta cells (MIN6 cells) [4, 5]. The expression of miR-24 was decreased response to high glucose culture condition in endothelial cells [4] but was reported upregulated by glucose and palmitate in beta cells [6].

miR-24 was identified targeting multiple 3′UTR sites and genes, directly regulating von Willebrand Factor (VWF) expression and related enzymes or proteins involved in the process of VWF synthesis, maturation and secretion [4]. These lend insight into miR-24-based coordinate regulation of VWF and provide a mechanism for the high plasma VWF levels and thrombotic complications observed with Type 2 diabetes. Thrombotic cardiovascular diseases is the leading cause of mortality among diabetic patients. Of particular interest is miR-24. In the heart, miR-24 has been reported to target BCL2L11 (BIM) and junctophilin-2 in cardiomyocyte, and targeting transcription factor GATA2, p21-activated kinase PAK4 and eNOS in endothelial cells [7]. In the vascular system, miR-24 was found to target chitinase 3-like 1 to limit vascular inflammation and matrix metalloproteinase-14 in macrophage to retard atherosclerotic plaque progression [7]. However, all of these findings are based on *in vitro* cultured cells and animal studies without an association with cardiovascular risk in human studies. Several studies have reported contrasting results in mouse myocardial infarction models. Some groups suggested that inhibition of miR-24 had beneficial therapeutic effects on myocardial infarction, whereas others supported improved recovery after myocardial infarction with miR-24 mimic therapy [7]. In line with these later studies, we identified decreased miR-24 in plasma and the heart under diabetic conditions [4]. Theoretically, miR-24 replacement therapy for diabetes should improve the heart functions and prevent cardiovascular complications. Our recent studies established the causality of diabetes and miR-24, and presented a novel mechanism for miR-24 down-regulation through hyperglycemia-induced activation of c-Myc [4]. Whether insulin resistance and hyperinsulinemia also regulate the expression or stability of miR-24 in diabetes remains unclear. Our unpublished data suggests that intensively insulin treatment for diabetic patients may contribute to diabetic thrombosis through miR-24. On the other hand, miRNAs (including miR-24) inactivation in β-cells of adult mice results in a striking diabetic phenotype [5]. These suggest that diabetic impaired miR-24 expression may feedback aggravate the diabetic conditions. In contrast, another recent study implicated miR-24 in the suppression of two maturity-onset diabetes of young (MODY) genes (HNF1α and NeuroD) [6]. miR-24 was found an increase in islets in the HFD-fed mice and db/db mice. But our unpublished results suggest that both aging and diabetes severity influence the miR-24 expression and downstream regulatory networks that fine-tune insulin expression and secretion in beta cells. That also partially explains the inconsistent cellular phenotype: knockdown of miR-24 did not affect the insulin expression in MIN6 cells but downregulated insulin in primary cultured islets [5]. In summary, miR-24 has multiple targets in beta cells, cardiomyocytes, endothelial cells, and macrophages, all of which are involved in diabetic complications. In turn, miR-24 itself is feedback regulated by glucolipotoxicity and impaired insulin pathways.

For miR-24-based therapeutics, large-animal studies are required but miR-24 has not been studied in large-animal models to date. Based on our recent findings that decreased miR-24 in diabetes increases the risk of diabetic thrombotic and vascular complications, miR-24 replacement therapy seems to be promising for diabetic patients. However, such miR-24 therapeutics also involve challenges as observations of miR-24 upregulation in cancers. The delivery dose and time should be well controlled. Another challenge is to achieve overexpression and cell-specific delivery of miR-24, which has divergent effects in endothelial cells versus cardiac cells. Nevertheless, the use of miR-24 mimics or inhibitors to control the diabetic pathophysiological processes offers new therapeutic opportunities.
